# Mortuary Statistics and Meteorological Report

**Published:** 1880-06

**Authors:** 


### MAY REPORT OF DEATHS AND INTERMENTS IN BALTIMORE

| CAUSE OF DEATH. | No. | CAUSE OF DEATH. | No. | CAUSE OF DEATH. | No. |
|---|---|---|---|---|---|
| Anemia | 1 | Dysentery | 4 | Malnutrition | 1 |
| Aneurism of Aorta | 1 | Endocarditis | 1 | Marasmus | 12 |
| Angina Pectoris | 1 | Epilepsy | 4 | Measels | 1 |
| Apoplexy | 22 | Erysipelas | 4 | Meningitis | 22 |
| Apynamic | 2 | Embolism | 2 | " Tubercular | 17 |
| Asphyxia | 1 | Exhaustion | 1 | " Cerebro Spinal | 6 |
| Asthma | 3 | Exposure | 1 | Oedema of Lungs | 1 |
| Asthenia | 4 | Fever Catarrh | 1 | " Brain | 1 |
| Bright's Disease | 5 | " Intermittent | 1 | Obstruction of Liver | 1 |
| Burns | 1 | " Puerperal | 1 | Old Age | 23 |
| Capillary Bronchitis | 5 | " Remittent | 3 | Paralysis | 11 |
| Cancer Abdomen | 1 | " Scarlet | 31 | Pneumonia | 29 |
| " Breast | 3 | " Typhoid | 10 | " Typhoid | 4 |
| " Bowels | 1 | " Typhus | 2 | " Pleuro | 1 |
| " Liver | 2 | " Malarial | 1 | Poison | 3 |
| " Face | 1 | Fracture Neck | 1 | Premature Birth | 20 |
| " Rectum | 1 | " Thigh | 1 | Prostration from Parturition | 1 |
| " Ventricular | 1 | " Tibris and Fibula | 2 | Prolonged Labor | 3 |
| " Uterus | 7 | " Skull | 3 | Puerperal Metritis | 2 |
| " Stomach | 6 | Gastro Enteritis | 6 | " Mania | 1 |
| Casualties | 6 | Goitre | 1 | Retention of Urine | 1 |
| Catarrh Gastro Intestinal | 2 | Gastric Hyperæmia | 1 | Pyæmia | 1 |
| " " | 2 | Gall Stone Colic | 1 | Rheumatism Inflammatory | 4 |
| " Senile | 1 | Haematurea | 1 | Scrofula | 5 |
| Cholera Infantum | 45 | Hernia, Strangulated | 1 | Softening of Brain | 3 |
| " Morbus | 2 | Hemorrhage of Bowels | 1 | Septicæmia | 3 |
| Cirrhosis of Liver | 2 | " Brain | 1 | Spine Bifada | 1 |
| Congestion of Brain | 13 | " Lungs | 1 | Stricture Intestines | 3 |
| " Kidneys | 1 | " Uterus | 1 | Sun Stroke | 1 |
| " Lungs | 2 | Hæmoptycis | 1 | Suicide | 1 |
| Consumption of Lungs | 115 | Hetun Exhaustion | 1 | Syphilis | 5 |
| Convulsions | 60 | Hydrothorax | 1 | Tabes Mesenterica | 1 |
| ' Puerperal | 4 | Inanition | 15 | Tetanus | 2 |
| Croup | 11 | Insanity | 2 | Trismus Nascentium | 3 |
| Cyanosis | 2 | Inflammation of Bladder | 2 | Tumor Stomach | 1 |
| Dentition | 5 | " Bowels | 7 | " Encephaloid | 1 |
| Delirium Tremens | 1 | " Stomach | 4 | " Neck | 1 |
| Diabetes | 1 | " Kidneys | 3 | Ulcer Bowels | 1 |
| Dia rhœa | 8 | " Larynx | 1 | " Stomach | 1 |
| Diphtheria | 18 | " Peritoneum | 5 | Unknown Adult | 1 |
| Disease of Brain | 3 | " Pleura | 1 | " Infantile | 3 |
| " Heart | 29 | Intussusception | 1 | Whooping Cough | 15 |
| " Spine | 1 | Intemperance | 4 | | |
| Dropsy (Gen'l) | 3 | Lithotomy | 1 | | 779 |
| Drowned | 7 | Meteorrhagia | 1 | | |

THE DEATHS REGISTERED IN THE MONTH INCLUDE-

| | | | |
|---|---|---|---|
| Under 1 year | 240 | Between 20 and 30 " | 64 |
| Between 1 and 2 years | 53 | " 30 and 40 " | 56 |
| " 2 and 5 years | 53 | " 40 and 50 " | 60 |
| | | " 50 and 60 " | 57 |
| Under 5 years | 346 | " 60 and 70 " | 45 |
| Between 5 and 10 years | 52 | " 70 and 80 " | 38 |
| " 10 and 15 " | 16 | " 80 and 90 " | 14 |
| " 15 and 20 " | 27 | " 90 and 100 " | 2 |

NATIVITY, ETC.

| | |
|---|---|
| United States, White | 486 |
| Colored | 170 |
| Foreign | 123 |
| | 779 |
| Still Birth | 55 |

Among the decedents were 58 above the age of 70 years, whose combined ages were 4,500 years, averaging 77 years, 6 months.

Estimated Population, 393,796. White, 338,384. Colored, 55,412. Annual Death Rate during the month, whole population, per 1,000—23.72. White, 21,62. Colored, 37.09.

By Order: A. R. CARTER, Secretary.

JAMES A. STEUART, M. D., Commissioner of Health and Registrar.

U. S. SIGNAL OFFICE, BALTIMORE, MD., June 1, 1880.

### METEOROLOGICAL SUMMARY FOR THE MONTH OF MAY, 1880.

#### PRESSURE.

Mean Monthly Barometer - - - - - 30.082 inches.
" " " for past 10 years 30.023 inches.
Highest Barometer - - - - - - 30,409 inches, on the 15th.
Lowest " - - - - - - - 29,818 inches, on the 6th.
Monthly Range of Barometer - - - - - 0,591 inches.

#### TEMPERATURE.

Mean Monthly Thermometer - - - - 31.1 degrees.
" " " for past 10 years 65.1 degrees.
Highest Thermometer - - 93 degrees on the 27th. Monthly Range - - 55 degrees.
Lowest " - - - 38 " " 1st. Mean Daily Range - 19.4 "

#### WIND, ETC.

Prevailing Direction of Wind, South.

Mean Velocity of Wind, 6.4 miles per hour.

Highest Velocity of Wind, 20 " "

Total Amount of Rainfall, 1.23 inches.

Number of Days on which Rain fell, 6.

Mean Humidity, 56.6 per cent.

ROBERT SEYBOTH, in charge.

